# Comparison of matrix frequency-doubling technology perimetry and standard automated perimetry in monitoring the development of visual field defects for glaucoma suspect eyes

**DOI:** 10.1371/journal.pone.0178079

**Published:** 2017-05-18

**Authors:** Rongrong Hu, Chenkun Wang, Lyne Racette

**Affiliations:** 1 Department of Ophthalmology, First Affiliated Hospital, College of Medicine, Zhejiang University, Hangzhou, Zhejiang, China; 2 Indiana University, Eugene and Marilyn Glick Eye Institute, Indianapolis, Indiana, United States of America; 3 Indiana University, Fairbanks School of Public Health, Indianapolis, Indiana, United States of America; Bascom Palmer Eye Institute, UNITED STATES

## Abstract

**Background:**

Perimetry is indispensable for the clinical management of glaucoma suspects. Our goal is to compare the performance of standard automated perimetry (SAP) and Matrix frequency-doubling technology (FDT) perimetry in monitoring the development of visual field (VF) defects in glaucoma suspect eyes.

**Methods:**

Longitudinal data of paired SAP and FDT from 221 eyes of 155 glaucoma suspects enrolled in the Diagnostic Innovations in Glaucoma Study or the African Descent and Glaucoma Evaluation Study were included. All eyes had glaucomatous optic neuropathy or ocular hypertension, but normal SAP and FDT results at baseline. The development of glaucomatous VF defects was defined as the presence of a cluster of ≥ 3 (less conservative) or ≥ 4 (more conservative) locations confirmed on ≥ 2 additional consecutive tests. Risk factors for the development of VF defects were analyzed by COX proportional hazard models. After conversion into common logarithmic units, the rates of change of global VF indices were fitted with linear mixed models.

**Results:**

FDT detected more eyes that developed VF defects than SAP using the less conservative criterion, and no significant difference was observed using the more conservative criterion. For those eyes detected by both SAP and FDT, FDT detected the development of VF defects either earlier than SAP or simultaneously in most cases. Baseline structural measurements were not significantly associated with an increased risk for the development of glaucomatous VF defects on either SAP or FDT. Older age was significantly associated with the development of VF defects on FDT but not on SAP. Both SAP and FDT detected a progressing worsening trend of pattern standard deviation over time with a similar rate of change between these test types.

**Conclusions:**

Matrix FDT would be useful to monitor the onset of VF defects in glaucoma suspects and may outperform SAP in the early stage of glaucomatous VF damage.

## Introduction

Despite the rapid advancement in imaging technologies, perimetry remains indispensable to detect the presence of glaucoma or its progression in clinical practice. White-on-white standard automated perimetry (SAP), which is not selective for a particular ganglion cell type, has been the reference standard to detect glaucomatous visual field (VF) damage. Frequency-doubling technology (FDT) perimetry is an alternative type of perimetry that has received wide interest. FDT was designed to target the magnocellular pathway [1] and recent studies show that other types of retinal ganglion cells and cortical factors may also mediate the perception of FDT stimuli [2–4].

Matrix FDT, the second generation of FDT, increases the spatial resolution of the test by using a 24–2 testing pattern similar to SAP and has a fairly short (4–5 minutes) test duration compared to SAP [5]. Unlike SAP, measurement variability does not increase with the worsening of VF sensitivity for FDT [6–11], which is advantageous to monitor the development of glaucomatous VF defects or its progression. Cross-sectional studies have shown Matrix FDT is comparable to or better than SAP in its discriminatory power for glaucomatous VF defects [12–16]. Less is known, however, about the ability of Matrix FDT to detect the progressive changes in eyes with a healthy status at baseline. The results of several recent longitudinal studies have not been conclusive in determining the performance of FDT compared to SAP in detecting progression [17–21]. The goal of the present study is to compare Matrix FDT and SAP in their ability to monitor the development of VF defects in a cohort of glaucoma suspect eyes without VF abnormality at baseline.

## Materials and methods

### Study cohort

The study included 221 eyes of 155 subjects selected from the Diagnostic Innovations in Glaucoma Study (DIGS) and the African Descent and Glaucoma Evaluation Study (ADAGES), which have been described in detail elsewhere [22]. In brief, these longitudinal studies were prospectively designed to assess structure and function in glaucoma. These multicenter studies were approved by all appropriate Institutional Review Boards, adhered to the tenets of the declaration of Helsinki for research involving human subjects, and were performed in conformity with the Health Insurance Portability and Accountability Act.

All subjects underwent a full ophthalmic examination and had open angles, best-corrected acuity of 20/40 or better, spherical refraction within 5.0 diopters, and cylinder correction within 3.0 diopters. Subjects were excluded if they had a history of intraocular surgery (except for uncomplicated cataract surgery); secondary causes of elevated intraocular pressure (e.g., iridocyclitis, trauma); other systemic or ocular diseases known to affect the VF (e.g., pituitary lesions, demyelinating diseases, human immunodeficiency virus positive or acquired immune deficiency syndrome, or diabetes); medications known to affect VF sensitivity; an inability to perform VF examinations reliably or life-threatening diseases.

### Inclusion criteria for the present study

All eyes included in this study were glaucoma suspects, defined as having documented glaucomatous optic neuropathy by stereophotographs or documented ocular hypertension [22], but without VF defects on SAP and Matrix FDT at the baseline of the present study. Glaucomatous optic neuropathy was defined as evidence of excavation, neuroretinal rim thinning or notching, localized or diffuse retinal nerve fiber layer defect, or a between-eye asymmetry of the vertical cup-disc ratio more than 0.2 [22]. Each photograph was graded by 2 certified graders independently according to a standard protocol using standard photographs as reference in a masked manner and in cases of disagreement, a third senior grader adjudicated [22].

All eyes therefore had normal VF that did not meet the minimum criteria for glaucomatous VF damage on both SAP and Matrix FDT. These criteria were defined as having a glaucoma hemifield test “outside normal limits”, or pattern standard deviation (PSD) at a P<5% level, or a cluster of three non-edge locations worse than a P level of 5% with at least one worse than a P level of 1% on the pattern deviation plot (PDP); and abnormality had to be confirmed on an additional test of the same type [23]. All eyes had at least 5 visits, each of which included one SAP and one Matrix FDT test that were taken within a 30-day window. A minimum of 3 months separated each of the consecutive visits. During the follow-up, each subject was treated or observed without treatment at the discretion of the attending ophthalmologists. All eyes were required to have a confocal scanning laser ophthalmoscopy image taken within three months of the baseline VF tests. All data were collected between 2003 and 2014 and individual information of included subjects cannot be identified.

### Visual field tests: SAP and Matrix FDT

The SAP tests were taken with the 24–2 pattern and Swedish interactive thresholding algorithm on the Humphrey Field Analyzer (Carl Zeiss Meditec, Dublin, California, USA). The FDT tests were taken with the 24–2 pattern and Zippy Estimation by Sequential Testing thresholding algorithm on the Humphrey Matrix FDT Perimeter (Carl Zeiss Meditec Inc., Dublin, CA) using Welch-Allyn technology. All visual fields were evaluated by the Visual Field Assessment Center at the Department of Ophthalmology, University of California, San Diego [24]. Only reliable visual fields, defined as ≤ 33% fixation losses, false-negative responses and false-positive responses, were included. Visual fields with the presence of artifacts (e.g., lid and lens rim artifacts) were excluded.

To determine whether a given eye developed glaucomatous VF defects, two criteria were used, which differed in their level of conservatism. Both were applied to SAP and Matrix FDT VF tests. First, the less conservative criterion required the presence of at least 3 adjacent locations in the same hemifield on the PDP, all at a P<5% level or worse with at least one location at a P<1% level or worse. A VF defect was identified when this cluster (using the same definition) was confirmed on at least two additional consecutive tests. Three tests were therefore required to identify a visual field defect. The more conservative criterion was based on Liu et al [20]. It required the presence of at least 4 adjacent locations in the same hemifield on the PDP, all at a P<5% level or worse with at least one location at a P<1% level or worse. This cluster (using the same definition) had to be confirmed by at least two additional consecutive tests.

### Baseline structural measures

Baseline structural measurements were taken by the confocal scanning laser ophthalmoscopy with the Heidelberg Retina Tomograph II (HRT II, software version 3.1, Heidelberg Engineering, Heidelberg, Germany). The HRT software acquires three individual images for each eye during the initial scanning, from which it automatically computes a mean topography image. An experienced technician outlined the optic disc margin on the mean topography image while viewing simultaneous stereophotographs of the optic disc [22]. Images with mean pixel height standard deviation more than 50 μm were excluded [25].

### Statistical analyses

The McNemar test was used to compare the proportion of eyes that developed glaucomatous VF defects on FDT and SAP. The agreement between FDT and SAP in identifying eyes that developed VF defects was calculated with the κ statistics. Agreement can be poor (κ value < 0.0), slight (0.01 to 0.20), fair (0.21 to 0.40), moderate (0.41 to 0.60), substantial (0.61 to 0.80), or almost perfect (0.81 to 1.0) [26]. Kaplan-Meier curves were used to analyze the difference between test types in the time of detection of glaucomatous VF defects. The survival probabilities on FDT and SAP were compared using Breslow tests.

Risk factors associated with the development of glaucomatous VF defects were analyzed by COX proportional hazard models. Separate COX proportional hazard models were fitted for FDT and SAP using the results based on the less conservative criterion. In eyes that developed VF defects, the time to the appearance of the defect was used. For eyes that did not develop VF defects, time to the last follow-up was used (censored observations). Rim area (RA), average retinal nerve fiber layer (RNFL) thickness, and cup-disc area ratio measured by HRT, and age at baseline were considered as independent variables in each model. The correlation between two eyes of the same subject was accounted by the maximum partial likelihood estimates under an independent working assumption using a robust sandwich covariance matrix estimate [27].

Longitudinal global VF indices [mean deviation (MD) and PSD] were fitted by linear mixed models. In order to have a common scale for the modeling, we converted the results obtained from the perimeters from decibels (dB) to log_10_ units using the approach outlined by Sun et al [28, 29]. For SAP, Weber contrast is used, which is the luminance increment divided by the mean luminance; for FDT, this is equivalent to Michelson contrast [28, 30]. One dB corresponds to 0.1 log_10_ unit change for SAP and to 0.05 log_10_ unit change for FDT [11, 28, 29, 31]. For SAP, the machine MD and PSD dB values can therefore be divided by 10 to obtain the log_10_ values. For FDT, the machine MD and PSD dB values can be divided by 20 to obtain the log_10_ values.

For the linear mixed models, follow-up time, test type, and their interaction were considered as the fixed effects. Random intercepts and slopes were included at the subject level. Hierarchical random intercepts were included at eye levels with two eyes nested within each subject. The Wald test was used to compare the main effect of test types and the rates of change of MD and PSD between FDT and SAP (interaction effect). SAP was considered as the reference type. A simulation study (repetition = 500) was conducted to evaluate the sample size requested for the linear mixed modeling. Assuming a balanced study design and all longitudinal measurements collected at year 0, 1, 2, 3, and 4, datasets were simulated based on the estimates of fixed effects, random intercept variance at both subject and eye levels, random slope variance, covariance between random intercept and random slope, and residual variance for MD and PSD. At an alpha of 0.05, the power analysis indicated that 100 subjects attained an empirical power of 99.8% for a rate of change of -0.01 log10 unit per year for MD, and an empirical power of 99.6% for a rate of change of 0.005 log10 unit per year for PSD.

All analyses were carried out in R (https://www.r-project.org) and SAS (version 9.4; SAS Institute, Inc., Cary, NC, USA). The R package visualFields [32] was used to process the VF data. P < 0.05 was considered statistically significant in all analyses.

## Results

At baseline, the mean age of the 155 glaucoma suspect subjects (221 eyes) included in the present study was 57.2 ± 8.9 years. One hundred and two subjects (65.8%) were female. Of the 221 eyes included in the study, 100 eyes (45.2%) had glaucomatous optic neuropathy and 121 (54.8%) eyes had ocular hypertension. Of the 89 fellow eyes that have not been included, 85 eyes had repeatable VF damage [23] on either or both test types at baseline, and 4 eyes were excluded due to unreliable VF results. The mean follow-up time of VF tests in each eye was 5.1 ± 1.8 years, with a median of 6 visits (ranging from 5 to 13 visits). The mean interval between visits in each eye was 11.0 ± 2.8 months. Table 1 shows the median, and 1st and 3rd quartiles of MD and PSD for SAP and FDT at baseline.

**Table 1:** Global indices of visual field at baseline.

	Median	First Quartile	Third Quartile
SAP MD, dB	-0.09	-0.87	0.79
SAP PSD, dB	1.46	1.30	1.65
FDT MD, dB	-0.29	-2.69	2.02
FDT PSD, dB	2.67	2.33	3.04

SAP, standard automated perimetry; FDT, frequency-doubling technology perimetry; MD, mean deviation; PSD, pattern standard deviation; dB, decibel.

**Fig 1:**
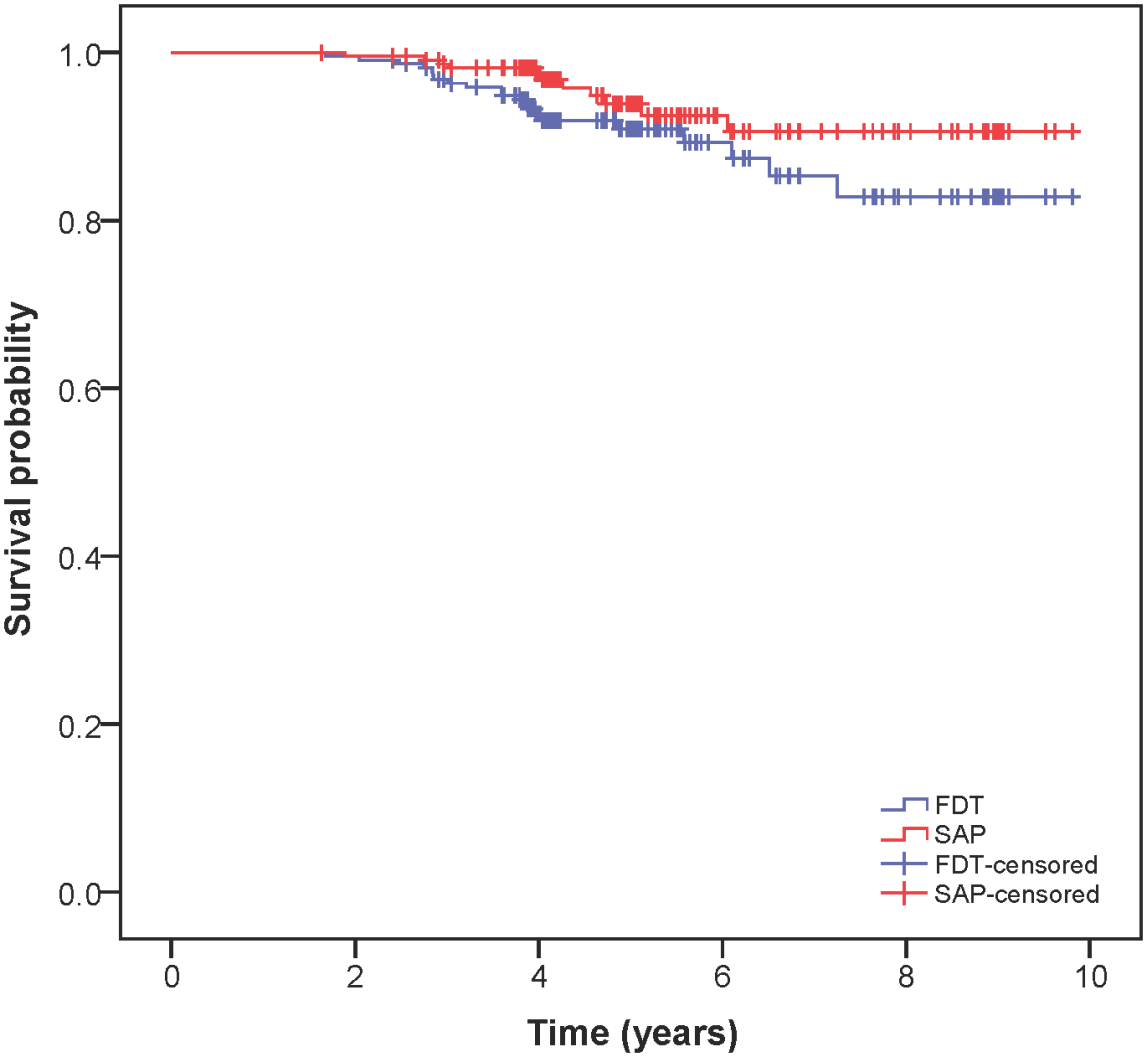
The Kaplan-Meier survival analysis for the development of glaucomatous VF defects. The Kaplan Meier survival curves show the survival probability of SAP and FDT for detection of VF development using the less conservative criterion. The hash marks represent the censored follow-ups for eyes that did not develop VF defects in the present study.

Using the less conservative criterion, 11 (5.0%) and 21 (9.5%) eyes developed glaucomatous VF defects on SAP and FDT, respectively. Using the more conservative criterion, 10 (4.5%) and 13 (5.9%) eyes developed VF defects on SAP and FDT, respectively. FDT identified significantly more eyes as developing VF defects than SAP (P = 0.02) based on the less conservative criterion. No significant difference was observed between SAP and FDT using the more conservative criterion (P = 0.51). Likewise, the survival probability of FDT for identifying the development of VF defects was significantly worse than SAP using the less conservative criterion (P = 0.04, Fig 1), and no significant difference was observed with the more conservative criterion (P = 0.31).

**Fig 2:**
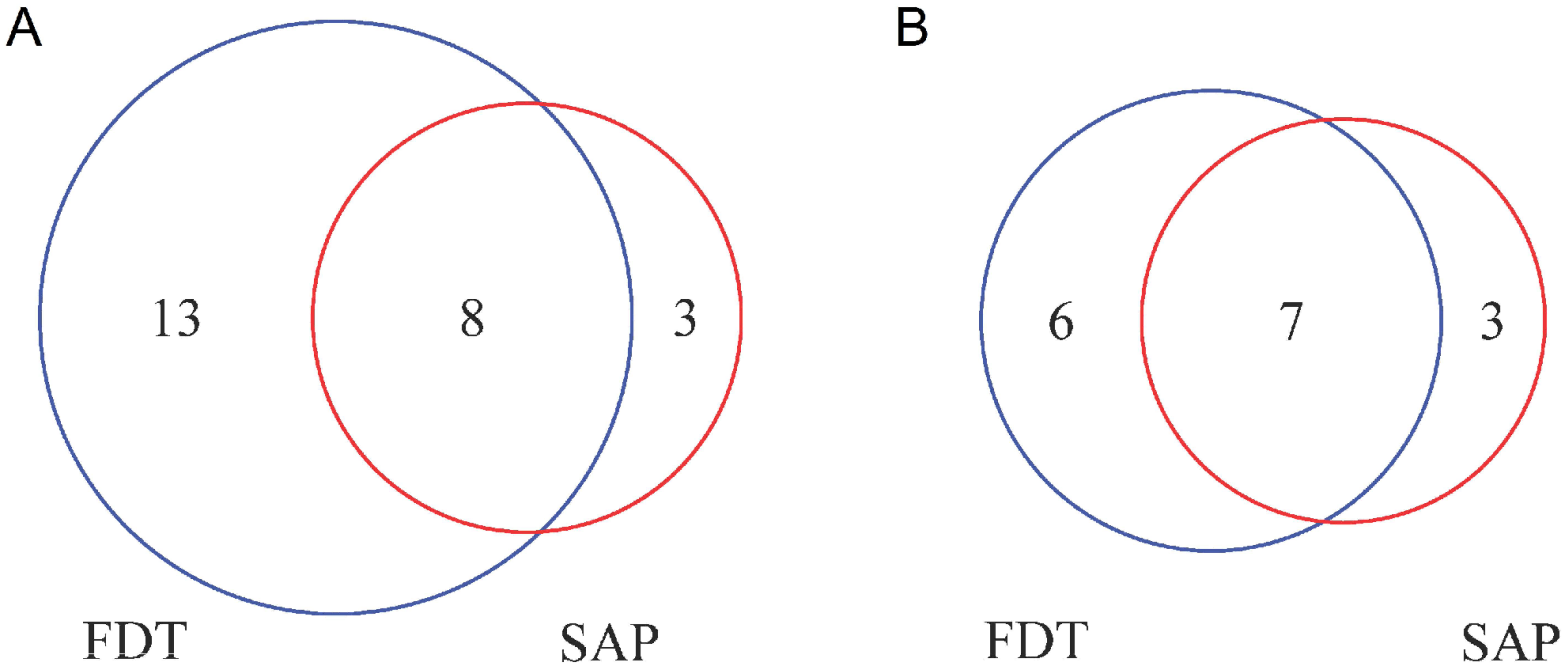
The numbers of eyes that developed glaucomatous VF defects. The Venn diagrams show the number of eyes with the development of glaucomatous VF defects detected by Matrix FDT and SAP using the less conservative criterion (A) and more conservative criterion (B).

Using the less conservative criterion, 8 eyes (3.6%) developed VF defects on both SAP and FDT, among which 1 eye was detected by SAP 11.5 months earlier than FDT, 5 eyes were detected by FDT 10.4 to 29.5 months earlier than SAP, and 2 eyes were detected by SAP and FDT at the same visit. Using the more conservative criterion, 7 eyes (3.2%) developed VF defects on both SAP and FDT, among which 1 eye was detected by SAP 10.5 months earlier than FDT, 5 eyes were detected by FDT 10.4 to 29.5 months earlier than SAP, and 1 eye was detected by SAP and FDT at the same visit. The agreement for detection of VF defects was moderate between SAP and FDT (κ = 0.47, ranging from 0.22 to 0.66 using the less conservative criterion, Fig 2A; κ = 0.59, ranging from 0.29 to 0.82 using the more conservative criterion, Fig 2B).

**Fig 3:**
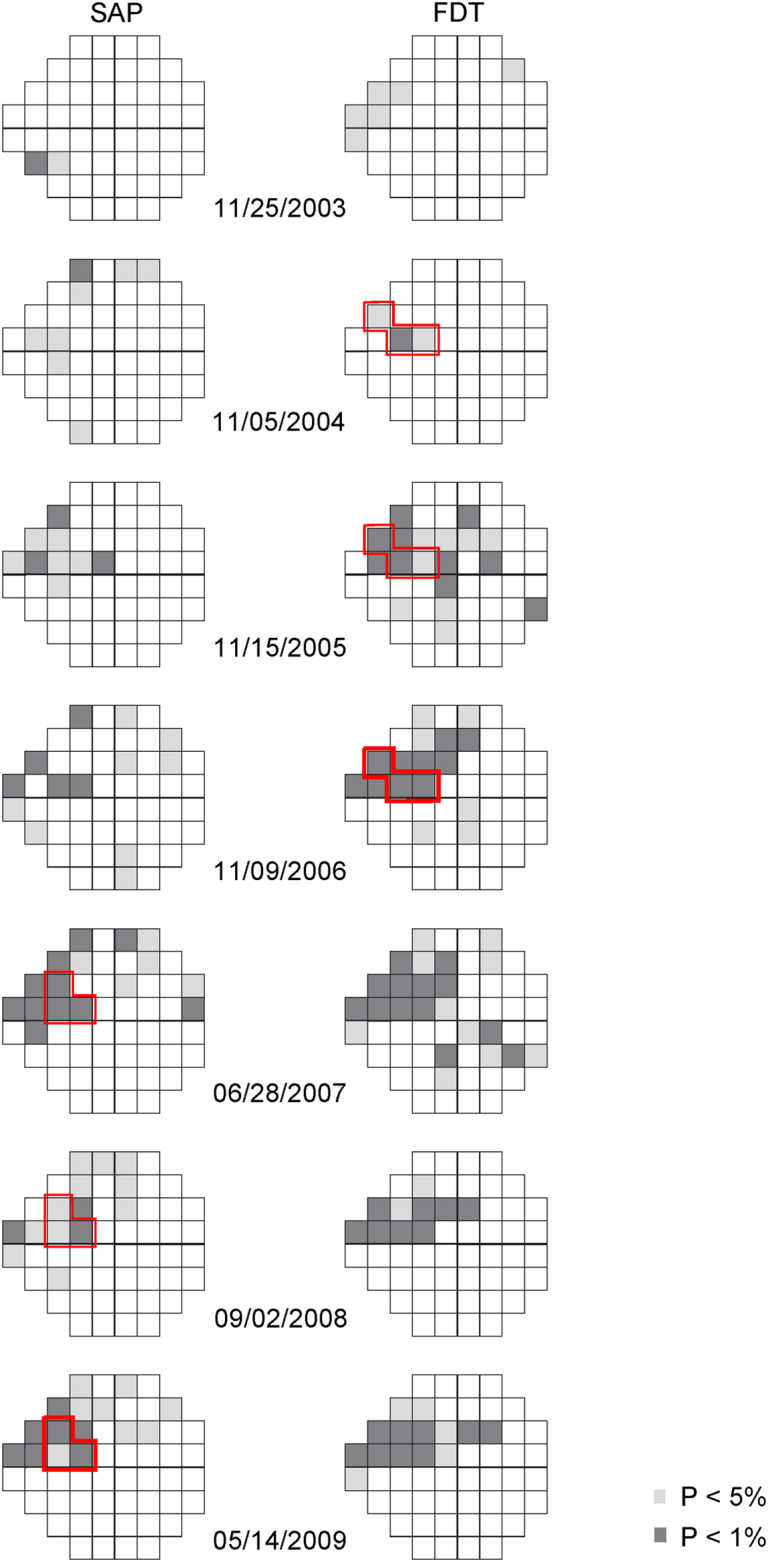
Longitudinal pattern deviation plots of an ocular hypertensive eye followed by SAP and Matrix FDT. The light-grey points and dark-grey points represent the visual field locations worse than a P level of 5% and 1% respectively. The red border marks the three locations on three consecutive tests used to identify the development of glaucomatous VF defects based on the less conservative criterion, respectively for SAP and FDT. The 3^rd^ test of the three consecutive tests is shown in thicker border.

Fig 3 shows a case of a patient with ocular hypertension followed by SAP and Matrix FDT in the present study. Using the less conservative criterion, FDT detected the development of a VF defect in the supero-nasal area, which was confirmed in November 2006. A progressing trend in the number and severity of abnormal locations was also observed. In contrast, more variability was observed with SAP results and based on the less conservative criterion, the confirmable VF defect in the same area was established until the end of the follow-up period.

The baseline HRT measurements (RA, RNFL thickness, and cup-disc area ratio) were not significantly associated with an increased risk for the development of glaucomatous VF defects on either SAP or FDT (Table 2). Age at baseline was significantly associated with the development of VF defects on FDT, with a hazard ratio of 1.13 for each year older in age on FDT (P = 0.001), but not on SAP (P = 0.09). The baseline HRT measurements and age data used in the COX proportional hazard models were included in the S1 File.

**Table 2:** Cox proportional hazard models for prediction of visual field development.

Variables	SAP				FDT			
	Hazard Ratio	P value	Lower (CL)	Upper (CL)	Hazard Ratio	P value	Lower (CL)	Upper (CL)
**Rim area, mm^2^**	1.61	0.73	0.11	23.39	0.16	0.12	0.02	1.59
**Average RNFL thickness, 10μm**	0.91	0.09	0.81	1.02	0.99	0.71	0.92	1.06
**Cup-disc area ratio, 0.1**	1.31	0.32	0.77	2.25	0.99	0.95	0.67	1.47
**Age, y**	1.08	0.09	0.99	1.18	1.13	0.001	1.05	1.22

SAP, standard automated perimetry; FDT, frequency-doubling technology perimetry; CL, confidence limit; RNFL, retinal nerve fiber layer; y, years.

Table 3 shows the results from multilevel linear mixed model with MD and PSD (expressed in a common log_10_ unit) as dependent variables, respectively. In the present cohort, the rate of change of PSD was 0.005 log_10_ unit per year (equivalent to 0.05 dB, P < 0.0001) on SAP and was 0.004 log_10_ unit per year (equivalent to 0.08 dB) on FDT; there was no significant difference in the rate of change of PSD between FDT and SAP (P = 0.12). No significant progressing trend was found for MD by either SAP or FDT. The dataset of longitudinal global VF indices fitted by the linear mixed models was included in the S2 File.

**Table 3:** Global trend analyses using linear mixed modeling (fixed effects).

Global index Effect	Mean deviation (MD: log_10_ unit)				Pattern standard deviation (PSD: log_10_ unit)			
	Estimate	P value	Lower (CL)	Upper (CL)	Estimate	P value	Lower (CL)	Upper (CL)
**Intercept**	-0.021	.	.	.	0.154	.	.	.
**FDT**	-0.017	0.001	-0.027	-0.007	-0.015	<.0001	-0.019	-0.011
**SAP**	reference	.	.	.	reference	.	.	.
**Time**	-0.002	0.301	-0.005	0.002	0.005	<.0001	0.003	0.006
Time*FDT	0.001	0.674	-0.002	0.003	-0.001	0.116	-0.002	0.000
Time*SAP	reference	.	.	.	reference	.	.	.

SAP, standard automated perimetry; FDT, frequency-doubling technology perimetry; CL, confidence limit.

## Discussion

Although the current version of the Matrix FDT allows for more direct comparison with SAP due to its similar testing grid, consensus has not been reached about its ability to monitor progressive VF loss [17–21]. In the present study, we compared the performance of the Matrix FDT and SAP in monitoring the development of VF defects in 221 glaucoma suspect eyes with normal SAP and FDT results at baseline. Based on the less conservative criterion (a cluster of 3 abnormal locations by 3 consecutive tests), FDT identified more eyes that developed glaucomatous VF defects compared to SAP. During the follow-up period, more eyes progressed on FDT compared to SAP using the survival analysis. For those eyes detected by both test types, FDT detected the development of VF defects either earlier than or simultaneously with SAP in most cases. The rate of change of PSD was similar for FDT and SAP. Our results suggest that the Matrix FDT may be useful to monitor glaucomatous VF damage in early disease.

We previously showed that FDT does not have significant benefits over SAP in monitoring glaucoma progression using pointwise linear regression analysis in a cohort of patients with primary open-angle glaucoma (median SAP MD of -2.25 dB at baseline) [21]. Likewise, using permutation of pointwise linear regression, Redmond et al [18] did not find evidence that FDT was more sensitive than SAP to identify VF progression in a cohort of patients diagnosed with glaucoma (mean SAP MD of -2.6 dB at baseline). FDT may, however, be more sensitive to VF changes in early disease, such as glaucoma suspect eyes with glaucomatous optic neuropathy or ocular hypertension (pre-perimetric glaucoma). Meira-Freitas et al [31], for example, showed that the rate of FDT PSD change was predictive of the development of SAP VF defects in glaucoma suspect eyes. The rate of SAP PSD change, however, was not predictive of the development of FDT defects in their study. Another study by Liu et al [20] showed that approximately 70% of glaucoma suspect eyes that developed VF defects were detected by FDT either prior to, or simultaneously with SAP (mean SAP MD of -1.27 dB at baseline). Hence, FDT may be of its highest value to monitor glaucomatous VF progression in the relatively early stages of the disease rather than in the later stages.

In the present study, FDT identified more eyes that developed glaucomatous VF defects than SAP using the less conservative criterion (a cluster of 3 abnormal locations by 3 consecutive tests), which was an event-based analysis. The rate of change on PSD was, however, similar for both FDT and SAP using the trend-based linear mixed modeling. Haymes et al [33] reported a similar finding using the first generation of FDT. They showed that FDT detected more progressing patients than SAP using glaucoma change probability analysis (event analysis), but that SAP detected more progressing patients than FDT using linear regression in the same study cohort.

Although there is no universally accepted method to identify VF progression, FDT may be better-suited for event analysis methods rather than trend analysis methods such as linear regression. Compared to SAP, the Matrix FDT has fewer discrete levels (0, 2, 3, 6, 7, 11, 12, 13, 18, 20, 23, 27, 32, 34, and 38 dB, while the step size of SAP is 1 dB). An underlying assumption of linear regression is that there is a trend of gradual, linear deterioration of VF sensitivity. FDT, with its larger steps, may therefore be at a disadvantage when compared to SAP to detect gradual changes in visual function. In comparison, the fewer discrete levels of FDT may have a smaller impact on its ability to identify change when event analysis is used. In addition, its more consistent test-retest variability [6–11] may give FDT an advantage to detect progression compared to SAP when event analysis is used.

Previous studies have shown that baseline HRT measurements can be used to predict the development of glaucomatous VF defects in suspect eyes defined as having ocular hypertension [34, 35]. In the present study, no baseline HRT measurement was significantly associated with an increased risk for the development of VF defects on either SAP or FDT. The disparity among the results of studies may be related to several factors, such as length of follow-up, inclusion criteria used to select study participants, definition of study end points, and so on. For example, the length of follow-up was shorter in the present study compared to previous studies [34, 35]. It is likely that more eyes would develop VF defects as the follow-up period increases and significant association between baseline HRT measurements and outcomes of VF defects would become apparent. Also we required the defects to be confirmed on two additional tests to maximize specificity, while previous studies [34, 35] only required defects to be confirmed on one additional test, which may lead to differences in the portions of eyes that developed VF defects. Our results are consistent with the notion that structural and functional measurements may each provide unique information for glaucoma [36–39]. Results from large clinical investigations [40–42] have identified older age as an independent risk factor for the development of glaucoma. For the present study cohort, older age was significantly associated with the development of glaucomatous VF defects on FDT, but not on SAP. Our findings affirm the usefulness of FDT in detecting the development of glaucomatous VF defects in suspect eyes. Higher baseline intraocular pressure (IOP) has been considered as another risk factor for the development of glaucoma [40–42]. Since a portion of our participants had begun IOP-lowering treatments at baseline based on the discretion of the attending ophthalmologists, we didn’t include baseline IOP for the analysis of risk factors.

Early detection of the onset of VF damage and its progression is paramount for the clinical management of glaucoma suspects. An unavoidable drawback of all studies investigating glaucoma progression, however, is the lack of a reference standard as no consensus has been reached to define progression [38]. For this reason, we used two levels of criteria to identify the development of VF defects and applied them to both SAP and FDT. Although these criteria may be arbitrary, the presence of three or four adjacent locations in the same hemifield on three consecutive tests would be enough to raise concern from a clinical perspective. While these criteria do not represent a reference standard, they have been used in previous studies [20, 43] and are clinically relevant. In the present study, FDT identified more eyes as developing VF defects compared to SAP using the less conservative criterion, while no significant difference between FDT and SAP was found using the more conservative criterion. It is possible that the more conservative criterion was not sensitive enough to allow the detection of early VF loss. Because of the lack of a gold standard and of a large enough sample of control eyes with longitudinal follow-up beyond the tests included in this study, we did not directly assess sensitivity and specificity in this study. Another limitation of the present study is that the development of glaucomatous VF defects was analyzed only based on the local VF defects and diffuse changes have not been considered, which is also a part of early glaucomatous VF damage [44].

While the results of both SAP and FDT are expressed in the dB units, their results cannot be directly compared because each test uses different stimuli and different measurement scales [11, 28–31]. In our study, for the linear mixed modeling, we expressed SAP and FDT data in a comparable scale by converting them into the common log_10_ values with the linear relationship. A recent study by Fredette et al [11] has also shown a linear 2:1 relationship exists between SAP and FDT data down to sensitivity values of 25 dB with SAP and 13 dB with FDT. Beyond this range, a nonlinear relationship exists between SAP and FDT, and an alternative conversion formula would be needed [11]. As the present study included glaucoma suspects with normal baseline VF results and only a small portion of them developed early glaucomatous VF defects, only a low percentage of our data came near the nonlinear range and we did not do the nonlinear conversion for those data. Specifically, 1,463 SAP and 1,463 FDT tests (52 locations * 1,463 VF tests = 76,076 locations) were available for analysis in the present study; 5.04% of SAP locations had sensitivity values below 25 dB (3,831 locations) and 1.97% of FDT locations (1,499 locations) had sensitivity values below 13 dB. Our conversion may also be defective as the Matrix FDT only provides 15 possible sensitivity values that are unevenly spaced, while the step size of SAP is 1 dB. In addition, several other factors may also complicate the comparison between SAP and FDT, such as different normative databases with different sample sizes and inclusion/exclusion criteria, different computation of VF indices, and so on. Further studies would be needed to determine the accurate correspondence of different measurements used on SAP and FDT.

In conclusion, our results demonstrate that the Matrix FDT is useful to monitor the onset of VF defects in glaucoma suspects during follow-up. Cross-sectional evidence has shown that glaucomatous VF defects detected by FDT when SAP is within normal range can be associated with RNFL thinning [45]. Further prospective studies to clarify its correlation with progressive structural change and to evaluate clinical decision-making based on the results of this test are needed.

## Supporting information

S1 File Baseline HRT measurements and age data used in the COX proportional hazard models. (CSV)

S2 File Longitudinal global VF indices fitted by the linear mixed models. (CSV)
